# Epidemiology of carcinoid neoplasms in Vaud, Switzerland, 1974–97

**DOI:** 10.1054/bjoc.2000.1394

**Published:** 2000-09-04

**Authors:** F Levi, V-C Te, L Randimbison, G Rindi, C La Vecchia

**Affiliations:** Registre vaudois des tumeurs, Institut universitaire de médecine sociale et préventive, Centre Hospitalier Universitaire Vaudois (CHUV), Chemin des Falaises 1, Lausanne, 1011, Switzerland; Unité d’épidémiologie du cancer, Institut universitaire de médecine sociale et préventive, rue du Bugnon 17, Lausanne, 1005, Switzerland; Anatomia Patologica 2, Spedali Civili, Piazza Spedali Civili 1, Brescia, 25124, Italy; Istituto di Ricerche Farmacologiche ‘Mario Negri’, Via Eritrea 62, Milano, 20157, Italy; Istituto di Statistica Medica e Biometria, Università degli Studi di Milano, Via Venezian 1, Milano, 20133, Italy

**Keywords:** carcinoid, epidemiology, incidence, registry, time trends, survival, intestinal, lung, second primary tumour

## Abstract

In Vaud, Switzerland, the incidence of carcinoids based on 218 malignant and 215 benign cases rose from 19.6/10^6^in 1974–85 to 28.2/10^6^in 1986–97, more so among males and malignant neoplasms. Lung was the commonest site for malignant and large intestine for benign carcinoids. Sixty-eight (16%) carcinoids had another neoplasm. © 2000 Cancer Research Campaign


*British Journal of Cancer* (2000) **83**(7), 952–955
© 2000 Cancer Research Campaign
doi: 10.1054/ bjoc.2000.1394, available online at http://www.idealibrary.com on IDEAL®

Short Communication

### Epidemiology of carcinoid neoplasms in Vaud, Switzerland, 1974–97

**F Levi[1,2], V-C Te[1], L Randimbison[2], G Rindi[3] and C La Vecchia[4,5]**

[1]Registre vaudois des tumeurs, Institut universitaire de médecine sociale et préventive, Centre Hospitalier Universitaire Vaudois (CHUV), Chemin des Falaises 1, 1011 Lausanne, Switzerland; [2]Unité d'épidémiologie du cancer, Institut universitaire de médecine sociale et préventive, rue du Bugnon 17, 1005 Lausanne, Switzerland; [3]Anatomia Patologica 2, Spedali Civili, Piazza Spedali Civili 1, 25124 Brescia, Italy; [4]Istituto di Ricerche Farmacologiche 'Mario Negri', Via Eritrea 62, 20157 Milano, Italy; [5]Istituto di Statistica Medica e Biometria, Università degli Studi di Milano, Via Venezian 1, 20133 Milano, Italy

**Summary** In Vaud, Switzerland, the incidence of carcinoids based on 218 malignant and 215 benign cases rose from $19.6/10^6$ in 1974–85 to $28.2/10^6$ in 1986–97, more so among males and malignant neoplasms. Lung was the commonest site for malignant and large intestine for benign carcinoids. Sixty-eight (16%) carcinoids had another neoplasm. © 2000 Cancer Research Campaign

**Keywords:** carcinoid; epidemiology; incidence; registry; time trends; survival; intestinal; lung; second primary tumour

Well differentiated neuroendocrine tumours or carcinoids include a spectrum of neoplasms originating in the neuroendocrine cells of the diffuse neuroendocrine system and sometimes producing a number of biologically active agents (Solcia et al, 2000b). Their clinical behaviour is hardly predictable on the basis of conventional clinico-pathological criteria (Läuffer et al, 1999). In addition, little or no information is available regarding their pathogenesis, with the notable exception of gastric carcinoids for which a role of hypergastrinemia has been suggested (Solcia et al, 2000b).

Most information on the epidemiology of neuroendocrine tumours comes from clinical and pathological series. A cancer registration-based dataset including 3382 malignant or 'uncertain behaviour' cases from England and Wales and 639 from Scotland over the period 1979–89 reported incidence rates around 1/100 000, with a female excess mainly in reproductive years, which could be partly but not totally accounted for better diagnosis and ascertainment (Newton et al, 1994). Further, a comprehensive report included 8305 cases of malignant carcinoid tumours registered in the Surveillance, Epidemiology and End Results (SEER) program of the National Cancer Institute (NCI), and in two previous NCI programs (Modlin and Sandor, 1997). In that series, carcinoid incidence rates were higher in females than in males, a noticeable proportion was associated with other neoplasms, the most frequent sites were the gastrointestinal tract, followed by lung, the overall five-year relative survival rate was over 50%, and the incidence of the disease had increased over the last 20 years.

We have updated our report on descriptive epidemiology and survival of carcinoids in the Swiss Canton of Vaud over the period 1974–97, the major population-based series from Continental Europe (Levi et al, 1993).

*Received 18 May 2000*
*Revised 15 June 2000*
*Accepted 15 June 2000*

*Correspondence to:* F Levi

The data were derived from the Vaud Cancer Registry datafile, which includes data concerning incident cases of malignant neoplasms and selected non-malignant or pre-cancerous lesions, diagnosed in the resident population of the Canton (whose population in 1990 was about 570 000 inhabitants; Levi et al, 1997). Information collected by the register includes general characteristics of the patient (age, sex), site and histological type of the tumour according to the standard International Classification of Diseases for Oncology (ICD-O, Ninth Revision; World Health Organization, 1976), and time of registration. Age-standardized rates were computed by the direct method, on the basis of the world standard population (Doll and Smith, 1987). Passive and active follow-up information is recorded, and each subsequent item of information concerning an already registered case is used to complete the corresponding record. Information coming from death certificates is routinely added to the incidence file. Information on vital status is derived, for 'apparently' non-deceased cases, from the registries of current residence.

The present series comprises 218 first malignant (i.e., locally infiltrating/metastastising; Levi et al, 1991, 1993) (118 males, 100 females), and 215 benign (77 males, 138 females) carcinoids registered from 1974 to 1997 in the resident population of the Canton. Only first occurrences were considered. In accord with the ICD-O classification (World Health Organization, 1976), the great majority of carcinoids diagnosed in the lung (ICD-O-M morphology code: 8240) were considered as malignant (fifth digit of ICD-O-M code: ≥3). Histological confirmation was 100%.

The vital status of each case considered for the present analysis was verified up to December 31, 1999. Twenty malignant cases (9%) and 22 benign cases (10%), discovered at autopsy, were not considered for survival analysis. Losses to follow-up were about 7%. Complete information on five-year survival was available for 343 (79%) uncensored cases and was computed starting from the date of histological confirmation. Relative survival rates (Ederer et al, 1961) were computed after allowance for the general lifetables of the canton (Levi et al, 1992).

**Table 1** Absolute numbers and age-adjusted incidence rates (rates per million, world standard population) of malignant and benign carcinoid tumours according to sex, calendar period and anatomical site. Vaud, Switzerland, 1974–1997

| Primary site | Males | | | | | | Females | | | | | | Males and Females | | | | | |
|---|---|---|---|---|---|---|---|---|---|---|---|---|---|---|---|---|---|---|
| | 74–85 | | 86–97 | | Total | | 74–85 | | 86–97 | | Total | | 74–85 | | 86–97 | | Total | |
| | *n* | Rate | *n* | Rate | *n* | Rate | *n* | Rate | *n* | Rate | *n* | Rate | *n* | Rate | *n* | Rate | *n* | Rate |
| *Malignant carcinoids* | | | | | | | | | | | | | | | | | | |
| Stomach | 1 | 0.3 | 3 | 0.7 | 4 | 0.5 | 0 | – | 1 | 0.2 | 1 | 0.1 | 1 | 0.1 | 4 | 0.5 | 5 | 0.3 |
| Small intestine | 11 | 2.3 | 31 | 5.8 | 42 | 4.1 | 10 | 1.2 | 20 | 3.5 | 30 | 2.4 | 21 | 1.6 | 51 | 4.4 | 72 | 3.1 |
| Colorectum | 4 | 1.3 | 10 | 2.6 | 14 | 1.9 | 9 | 2.7 | 10 | 2.2 | 19 | 2.4 | 13 | 2.0 | 20 | 2.3 | 33 | 2.1 |
| Lung | 12 | 3.2 | 25 | 5.8 | 37 | 4.6 | 15 | 3.5 | 22 | 3.6 | 37 | 3.5 | 27 | 3.3 | 47 | 4.7 | 74 | 4.0 |
| Other | 6 | 1.3 | 15 | 2.6 | 21 | 2.0 | 2 | 0.2 | 11 | 1.7 | 13 | 1.0 | 8 | 0.6 | 26 | 2.0 | 34 | 1.4 |
| Total, all sites | 34 | 8.4 | 84 | 17.6 | 118 | 13.1 | 36 | 7.7 | 64 | 11.1 | 100 | 9.4 | 70 | 7.7 | 148 | 13.9 | 218 | 10.9 |
| *Benign carcinoids* | | | | | | | | | | | | | | | | | | |
| Stomach | 2 | 0.4 | 4 | 1.0 | 6 | 0.7 | 5 | 0.6 | 3 | 0.1 | 8 | 0.4 | 7 | 0.5 | 7 | 0.6 | 14 | 0.6 |
| Small intestine | 15 | 3.1 | 17 | 3.1 | 32 | 3.1 | 4 | 0.3 | 8 | 0.9 | 12 | 0.7 | 19 | 1.5 | 25 | 1.9 | 44 | 1.7 |
| Colorectum | 13 | 3.2 | 23 | 7.2 | 36 | 5.2 | 50 | 15.0 | 55 | 14.8 | 105 | 14.9 | 63 | 9.2 | 78 | 11.0 | 141 | 10.1 |
| Lung | 1 | 0.1 | 0 | – | 1 | 0.1 | 3 | 0.7 | 1 | 0.2 | 4 | 0.5 | 4 | 0.4 | 1 | 0.1 | 5 | 0.3 |
| Other | – | – | 2 | 0.4 | 2 | 0.2 | 2 | 0.4 | 7 | 1.0 | 9 | 0.7 | 2 | 0.2 | 9 | 0.7 | 11 | 0.5 |
| Total, all sites | 31 | 6.8 | 46 | 11.7 | 77 | 9.2 | 64 | 17.2 | 74 | 17.1 | 138 | 17.1 | 95 | 11.9 | 120 | 14.3 | 215 | 13.1 |
| *Benign and malignant carcinoids* | | | | | | | | | | | | | | | | | | |
| Stomach | 3 | 0.7 | 7 | 1.7 | 10 | 1.2 | 5 | 0.6 | 4 | 0.3 | 9 | 0.5 | 8 | 0.7 | 11 | 1.0 | 19 | 0.9 |
| Small intestine | 26 | 5.4 | 48 | 9.0 | 74 | 7.2 | 14 | 1.6 | 28 | 4.4 | 42 | 3.1 | 40 | 3.1 | 76 | 6.3 | 116 | 4.8 |
| Colorectum | 17 | 4.5 | 33 | 9.8 | 50 | 7.1 | 59 | 17.7 | 65 | 17.0 | 124 | 17.3 | 76 | 11.2 | 98 | 13.4 | 174 | 12.2 |
| Lung | 13 | 3.3 | 25 | 5.8 | 38 | 4.6 | 18 | 4.3 | 23 | 3.8 | 41 | 4.0 | 31 | 3.7 | 48 | 4.8 | 79 | 4.3 |
| Other | 6 | 1.3 | 17 | 3.0 | 23 | 2.3 | 4 | 0.6 | 18 | 2.7 | 22 | 1.7 | 10 | 0.9 | 35 | 2.7 | 45 | 1.9 |
| Total, all sites | 65 | 15.2 | 130 | 29.3 | 195 | 22.4 | 100 | 24.8 | 138 | 28.2 | 238 | 26.5 | 165 | 19.6 | 268 | 28.2 | 433 | 24.0 |

Table 1 gives the distribution of 433 cases of carcinoids according to sex, site, calendar period and histological type. Overall, age standardized incidence rates for both sexes and all sites combined rose from $19.6/10^6$ in 1974–85 to $28.2/10^6$ in 1986–97. The rise was apparently larger in males (from 15.2 to $29.3/10^6$) than in females (from 24.8 to $28.2/10^6$), in the absence of any clear pattern across sites. Further, the upward trends over time were apparently larger for malignant (from 7.7 to $13.9/10^6$) than for benign (from 11.9 to $14.3/10^6$) neoplasms.

Colorectum was the most common site (*n* = 174, 40%), followed by small intestine (*n* = 116, 27%), lung (*n* = 79, 18%), and stomach (*n* = 19, 4%), (Table 1). Lung was the most common site for malignant carcinoids, and intestines (mainly colorectum) for benign ones, while less than 5% of benign carcinoids were outside digestive tract. Overall, 141 out of 174 (81%) tumours of the colorectum were benign, and 14 out of 19 (74%) gastric tumours. Conversely, only 44 out of 116 (38%) tumours of the small intestine were benign and only five out of 79 (6%) in the lung. The upward trends over time were somewhat larger for small intestines and other sites, but they were observed across all separate sites.

Figure 1 gives age-specific incidence rates for malignant, benign carcinoids, and separately for digestive tract and lung for malignant cases. The incidence rates rose with age both for malignant and benign cases, particularly for digestive tract (from stomach to rectum).

Table 2 gives survival rates for malignant carcinoids in two calendar periods 1974–85, and 1986–97. Five-year relative survival increased from 0.64 to 0.75 for males and from 0.73 to 0.83 for females and in both sexes, was highest for lung, followed by digestive tract.

Sixty-eight subjects (16% of all carcinoids; 35 malignant, 33 benign) had another either synchronous (i.e. diagnosed less than three months after the first primary tumour; *n* = 51) or metachronous (*n* = 17) malignant non carcinoid non cutaneous (excluding non-melanomatous skin) neoplasm. Two additional subjects were registered with two intestinal primary carcinoids (two malignant synchronous carcinoids, one of the small bowel and one of the caecum; one benign and one malignant metachronous rectal carcinoid in the other subject). Of the 51 synchronous tumours, 18 were in the digestive tract, nine in the lung, and 24 in various combinations of sites. Of the 17 metachronous tumours, only one was in the digestive tract, and among the remaining 16 second primary non carcinoid neoplasms two lung, five breast, two prostate, and one oral cavity, intestines, bladder, kidney, brain, lymphoid, and haemopoietic tissue. The number of metachronous neoplasms was however lower than the expected one (i.e., 17 cases observed versus 27.3 expected; standardized incidence ratio = 0.62, 95% confidence interval: 0.36–1.00).

The present work confirms, in a uniformly surveyed population, that carcinoids are rare – but not extremely rare – neoplasms, with overall age-standardized rates around 1/100 000 for both benign and malignant carcinoids. Malignant carcinoids were about 40% more common in males than in females, whereas benign ones were about two-fold more common in females (Rindi et al, 1996). This may reflect greater opportunities for diagnosis particularly for abdominal carcinoids in females following laparotomies in reproductive age or for other conditions (e.g. gallstones) more common in females (Thompson et al, 1985; Newton et al, 1994). An influence of sex hormones on (benign) carcinoids cannot be excluded, in view of the reported presence of oestrogen receptor proteins (Keshgegian and Wheeler, 1980). Female predominance has also

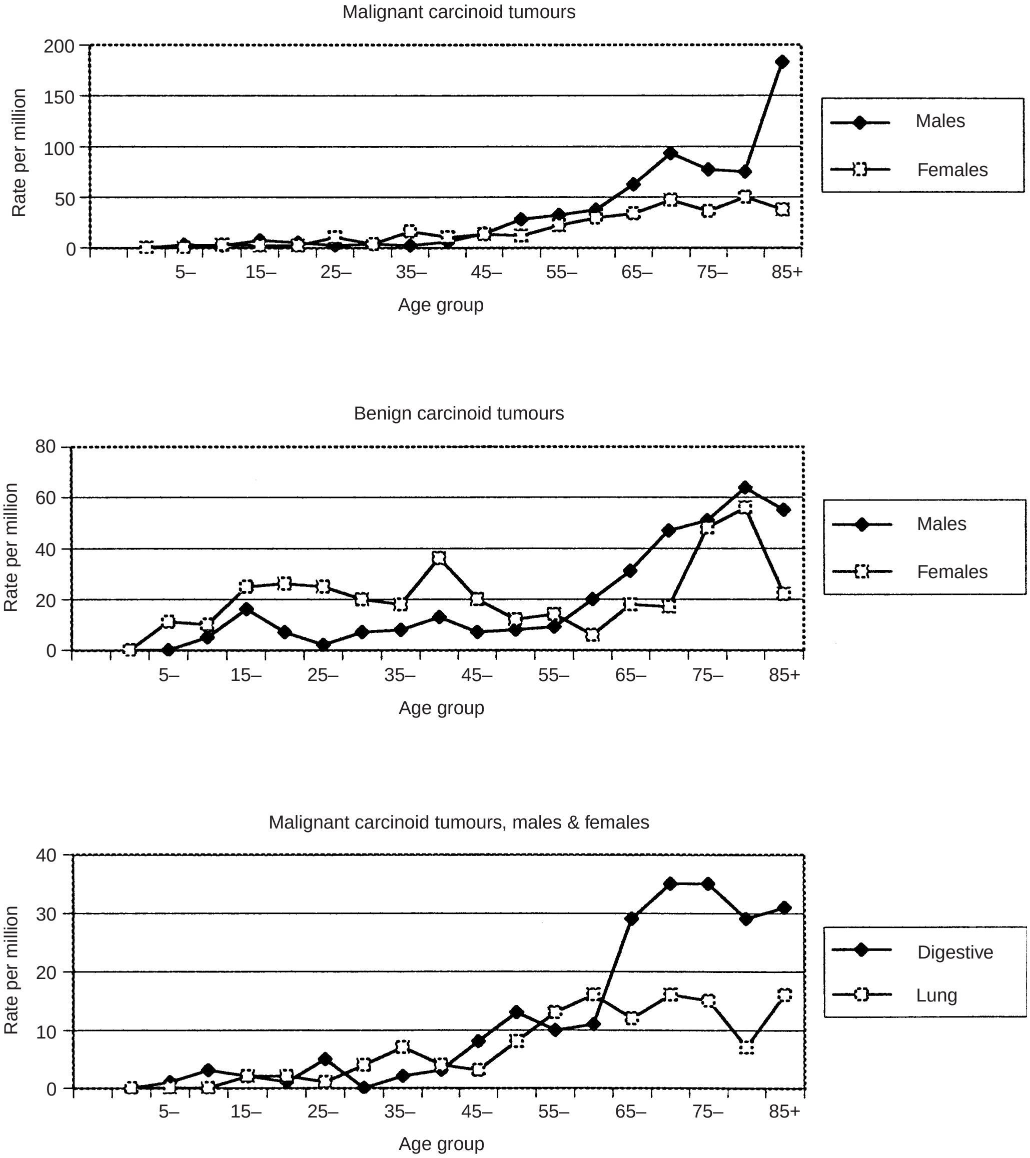

**Figure 1** Age-specific incidence curves (rates per million per years) by sex for malignant and benign carcinoids, and by site, both sexes combined, for malignant carcinoids been observed in an African rodent model of gastric carcinoids (Modlin et al, 1994; Modlin and Sander, 1997).

The site distribution of carcinoids confirms that carcinoids are commonest in the colorectum, followed by small intestine, lung, and stomach.

Benign tumours were observed mainly in the colon and stomach, as in pathological series (Fiocca et al, 1987; Rindi et al, 1996). This finding may well reflect the early symptomatic features of the frequently polypoid carcinoids of the upper and lower digestive tract, as well with their relatively straightforward investigation and treatment. Most tumours of the small intestine and lung were classified as malignant. Differences in the cell biology of tumours from different sites cannot be excluded.

Over the two calendar periods considered (1974–85 and 1986–97), incidence rates of benign carcinoids in both sexes combined increased by about 20%, whears malignant neoplasms almost doubled. The largest proportional rises in incidence were observed for stomach and small intestine. These upward trends may be related to diagnostic improvements during the last decade, following the widespread use of specific markers in pathology practice, and the introduction of somatostatin analogs for imaging procedures (Krenning et al, 1999; Solcia et al, 2000a). An influence of diagnostic improvements in malignant carcinoids is indicated by the observed increased survival over time. For gastric carcinoids, this rise in incidence would be consistent with the introduction and subsequent widespread use of powerful acid inhibitory drugs which stimulate gastric secretion (Mignon et al, 1987; Rindi et al, 1996). The latter has been shown in animal models to increase malignant gastric carcinoids (Larsson et al, 1986; Modlin et al, 1988, 1989; Läuffer et al, 1999). However, no information on drug use was available in the Vaud cancer register.

With reference to the age distribution, this study confirms (Newton et al, 1994; Modlin and Sandor, 1997) that the incidence of both benign and malignant carcinoids increases with age, like

 *© 2000 Cancer Research Campaign*

**Table 2** Five-year relative survival rates after malignant carcinoid tumour according to primary site, sex, and calendar period. Vaud, Switzerland, 1974–97

| | Calendar period | | | | | | | | |
|---|---|---|---|---|---|---|---|---|---|
| | 1974–85 | | | 1986–97 | | | 1974–97 | | |
| Primary site | Males | Females | Total | Males | Females | Total | Males | Females | Total |
| Digestive tract | 0.74 (0.13)[a] | 0.71 (0.12) | 0.72 (0.09) | 0.79 (0.08) | 0.86 (0.08) | 0.82 (0.06) | 0.78 (0.07) | 0.80 (0.07) | 0.79 (0.05) |
| Lung | 0.67 (0.15) | 0.83 (0.10) | 0.77 (0.09) | 0.83 (0.09) | 0.91 (0.09) | 0.86 (0.07) | 0.77 (0.08) | 0.86 (0.07) | 0.82 (0.06) |
| Other sites | 0.27 (0.18) | 0.68 (0.35) | 0.39 (0.17) | 0.49 (0.13) | 0.61 (0.16) | 0.54 (0.10) | 0.43 (0.11) | 0.63 (0.14) | 0.52 (0.09) |
| Total, all sites | 0.64 (0.09) | 0.73 (0.08) | 0.71 (0.06) | 0.75 (0.06) | 0.83 (0.06) | 0.79 (0.04) | 0.72 (0.05) | 0.80 (0.05) | 0.76 (0.04) |

[a] Standard errors of the rates are given in parentheses most epithelial neoplasms, the levelling off for benign ones above age 80 being partly or largely attributable to diagnostic difficulties in the elderly.

The overall survival rate of 76% is appreciably greater than the 50% reported in the SEER dataset. However, absolute rather than relative survival was given by the SEER dataset.

This study also confirms (Berner, 1993) that a substantial proportion carcinoids was associated with other synchronous neoplasms. This proportion is similar to that observed in the SEER database (13%; Modlin and Sandor, 1997). The lower than expected number of metachronous neoplasms suggests that at least part of the excess of second neoplasms in patients with carcinoids is attributable to careful diagnostic ascertainment at original diagnosis.

### ACKNOWLEDGEMENTS

The contribution of the Vaud Cancer Registry's staff is gratefully acknowledged.

### REFERENCES

Berner M (1993) Carcinoïdes digestifs et tumeurs malignes synchrones. *Schweiz Med Wschr* **123**: 594–599

Doll R and Smith PG (1987) Comparison between registries: age-standardized rates. In: *Cancer Incidence in Five Continents, Vol. IV*, JAH Waterhouse, CS Muir, K Shanmugaratham, J Powell, D Peacham and S Whelan (eds), Lyon, International Agency for Research on Cancer, IARC Scient Publ **42**: 634–637

Ederer F, Axtell MA and Cutler SJ (1961) The relative survival rate: a statistical methodology. *Natl Cancer Inst Monogr* **6**: 101–121

Fiocca R, Rindi R, Capella C, Grimelius L, Polak JM, Schwartz TW, Yanaihara N and Solcia E (1987) Glucagon, glicentin, proglucagon, PYY, PP and proPP-icosa-peptide immunoreactivites of rectal carcinoid tumours and related non-tumour cells. *Reg Pep* **17**: 9–29

Keshgegian AA and Wheeler JE (1980) Estrogen receptor protein in malignant carcinoid tumor. *Cancer* **45**: 293–296

Krenning EP, de Jong M, Kooij PPM, Breeman WAP, Bakker WH, de Herder WW, van Ejick CHJ, Kwekkeboom DJ, Jamar F, Pauwels S and Valkema R (1999) Radiolabeled somatostatin analogues for peptide receptor scintigraphy and radionuclide therapy. *Ann Oncol* **10**(Suppl. 2): S23–S29

Larsson H, Carlsson E, Mattsson H, Lundell L, Sundler F, Sundell G, Wallmark B, Watanabe T and Hakanson R (1986) Plasma gastrin and gastric enterochromaffin-like cell activation and proliferation. *Gastroenterology* **90**: 391–399

Läuffer JM, Zhang T and Modlin IM (1999) Review article: current status of gastrointestinal carcinoids. *Aliment Pharmacol Ther* **13**: 271–287

Levi F, La Vecchia C, Franceschi S and Te VC (1991) Morphologic analysis of digestive cancers from the Registry of Vaud, Switzerland. *Br J Cancer* **63**: 567–572

Levi F, Randimbison L, Te VC, Franceschi S and La Vecchia C (1992) Trends in cancer survival in Vaud, Switzerland. *Eur J Cancer* **28**: 1490–1495

Levi F, Randimbison L, Franceschi S and La Vecchia C (1993) Descriptive epidemiology of malignant carcinoids in the Swiss Canton of Vaud. *Int J Cancer* **53**: 1036–1037

Levi F, Te VC, Randimbison L and La Vecchia C (1997) Statistics from the Registry of the Canton of Vaud, Switzerland, 1988–92. In: *Cancer Incidence in Five Continents*, Vol VII. Parkin DM, Whelan S, Ferlay J, Raymond L, Young J. (eds). Lyon, International Agency for Research on Cancer. IARC Scien Pub **143**: 674–677

Mignon M, Lehy T, Bonnefond A, Ruszniewski P, Labeille D and Bonfils S (1987) Development of gastric argyrophil carcinoid tumours in a case of Zollinger-Ellison syndrome with primary hyperparathyroidism during long-term secretory treatment. *Cancer* **59**: 1959–1962

Modlin IM and Sandor A (1997) An analysis of 8305 cases of carcinoid tumors. *Cancer* **79**: 813–829

Modlin I, Zucker K, Zdon M, Sussman J and Adrian T (1988) Characteristics of the spontaneous gastric endocrine tumor of Mastomys. *J Surg Res* **44**: 205–215

Modlin I, Bilchik, Nilsson O and Goldenring J (1989) Enterochromaffin-like cell tumor induction in Mastomys by insurmontable H2 receptor blokade. *Res Clin Forums* **12**: 59–74

Modlin IM, Lawton GP, Tang LH, Geibel J, Abraham R and Darr U (1994) The Mastomys gastric carcinoid: aspects of enterochromaffin-like cell function. *Digestion* **55**(Suppl 3): 31–37

Newton JN, Swerdlow AJ, dos Santos Silva IM, Vessey MP, Grahame-Smith DG, Primatesta P and Reynolds DJM (1994) The epidemiology of carcinoid tumours in England and Scotland. *Br J Cancer* **70**: 939–942

Rindi GR, Bordi C, Rappel S, La Rosa S, Stolte M and Solcia E (1996) Gastric carcinoids and neuroendocrine carcinomas: pathogenesis, pathology and behavior. *World J Surg* **20**: 168–172

Solcia E, Kloppel G and Sobin LH (2000a) Histological typing of endocrine tumors. WHO Classification of Endocrine Tumours. Springer-Verlag, Berlin, 160

Solcia E, Rindi G, LaRosa S and Capella C (2000b) Morphological, molecular, and prognostic aspects of gastric endocrine tumors. *Microsc Res Tech.* **48**: 339–348

Thompson GB, Van Heerden JA, Martin JK, Schutt AJ, Ilstrup DM and Carney JA (1985) Carcinoid tumours of the gastrointestinal tract: presentation, management and prognosis. *Surgery* **98**: 1054–1062

World Health Organization (1976) International Classification of Diseases for Oncology, ICD-O. Geneva: World Health Organisation, p. 131

*© 2000 Cancer Research Campaign* 

